# Induction of Lupus T Cell Subset by Probiotics‐Matured Tolerogenic Dendritic Cells

**DOI:** 10.1002/fsn3.71283

**Published:** 2025-12-01

**Authors:** Maryam Ahmadi‐Khorram, Mahmoud Mahmoudi, Ramiar Kamal Kheder, Maryam Rastin, Alireza Hatami, Afsane Fadaee, Seyed‐Alireza Esmaeili

**Affiliations:** ^1^ Department of Nutrition, Faculty of Medicine Mashhad University of Medical Sciences Mashhad Iran; ^2^ Student Research Committee Mashhad University of Medical Sciences Mashhad Iran; ^3^ Immunology Research Center Mashhad University of Medical Sciences Mashhad Iran; ^4^ Immunology Department, Faculty of Medicine Mashhad University of Medical Sciences Mashhad Iran; ^5^ Medical Laboratory Science Department, College of Science University of Raparin Sulaymaniyah Iraq; ^6^ Department of Medical Analysis, Faculty of Applied Science Tishk International University Erbil Iraq

**Keywords:** dendritic cells, immune modulation, *Lactobacillus delbrueckii*, *Lactobacillus rhamnosus*, probiotics, regulatory T cells, systemic lupus erythematosus, T cell subset

## Abstract

Systemic lupus erythematosus (SLE) is a chronic autoimmune disease characterized by immune dysregulation, leading to tissue inflammation and damage. Tolerogenic dendritic cells (DCs) and regulatory T cells (Tregs) play critical roles in restoring immune balance, and their dysfunction is implicated in SLE pathogenesis. This study investigates the immunomodulatory effects of *Lactobacillus rhamnosus* and *Lactobacillus delbrueckii* on DC function and T cell subset differentiation in SLE patients. Peripheral blood mononuclear cells (PBMCs) were isolated from SLE patients and healthy controls. Mixed lymphocyte reactions (MLRs) were performed to evaluate the effects of DCs treated with *L. rhamnosus* , *L. delbrueckii* , or their combination on T cell differentiation. Expression of immune markers (FOXP3, TGF‐β, IL‐10, ROR‐γt, IL‐17, T‐bet, IFN‐γ, GATA3, IL‐4) was quantified using real‐time PCR, and Treg, Th1, and Th17 populations were assessed via flow cytometry. Probiotic‐treated DCs significantly increased Treg populations and FOXP3 and TGF‐β expression in SLE patients compared to healthy controls, with the combined probiotic treatment showing the most pronounced effect. Th17 and Th1 cell populations, along with ROR‐γt and IFN‐γ expression, were significantly reduced in probiotic‐treated groups, particularly in SLE patients. IL‐10 expression increased but did not reach statistical significance. *L. rhamnosus* and *L. delbrueckii* promote tolerogenic DC function, enhancing Treg induction and suppressing pro‐inflammatory Th17 and Th1 responses in SLE. The synergistic effect of combined probiotics suggests their potential as a complementary therapy for immune modulation in autoimmune diseases. Further clinical studies are needed to confirm their efficacy and safety in SLE management.

## Graphical abstract

Probiotic‐matured tolerogenic dendritic cells (DCs) from *Lactobacillus rhamnosus* and *L. delbrueckii* significantly enhanced regulatory T cell (Treg) induction via increased FOXP3 and TGF‐β in SLE patients. Combined probiotic treatment (P‐MIX) showed the strongest Treg expansion (*p* = 0.0004) and suppressed Th17/Th1 responses by reducing ROR‐γt and IFN‐γ. These findings suggest multi‐strain probiotics as a promising adjuvant therapy for restoring immune balance in systemic lupus erythematosus.

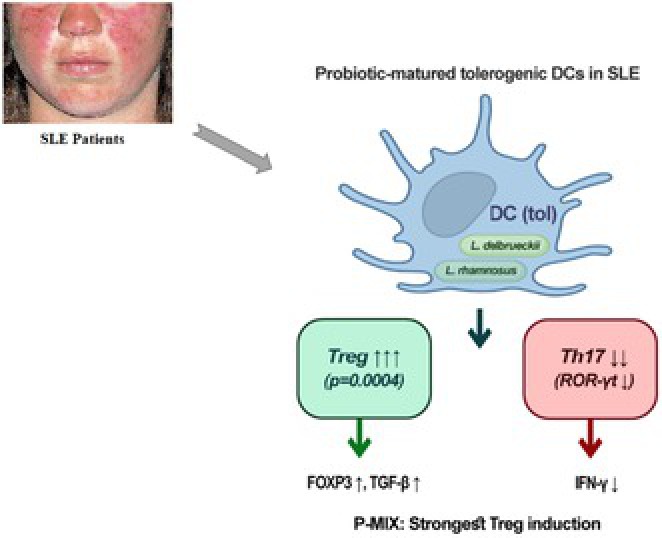

## 1 Introduction

Systemic lupus erythematosus (SLE) is a chronic condition marked by immune system dysfunction, resulting in widespread inflammation and damage to multiple organs (Bahadorian et al. 2024; Mahmoudi et al. 2023; Yazdanpanah et al. 2017). Its complex pathogenesis emerges from a dynamic interplay of genetic predispositions, epigenetic modifications, environmental triggers, and immunological abnormalities, which collectively undermine the body's immune homeostasis (Araki and Mimura 2024; Kim et al. 2025; Tsokos 2024). Dendritic cells (DCs), key antigen‐presenting cells that serve as a crucial link between innate and adaptive immunity, are instrumental in the pathology of SLE. Tolerogenic DCs, which enhance immune tolerance by secreting anti‐inflammatory cytokines like interleukin‐10 (IL‐10) and transforming growth factor‐beta (TGF‐β), promote the development and function of regulatory T cells (Tregs), essential for suppressing excessive immune responses (Mellman 2013; Sousa et al. 2017; Zong et al. 2024; Javanmardi et al. 2024; Sahebkar 2020; Esmaeili et al. 2021). In SLE, diminished Treg numbers and function, coupled with heightened pro‐inflammatory Th1 and Th17 cell activity, drive autoimmunity through elevated interferon‐gamma (IFN‐γ) and interleukin‐17 (IL‐17) production, exacerbating tissue damage (Khorasani et al. 2019; Dolff et al. 2011; Huang et al. 2024). Consequently, therapeutic strategies that enhance Treg activity while suppressing Th1 and Th17 responses hold significant promise for SLE management.

Emerging evidence highlights the gut microbiome as a key regulator of immune tolerance, with probiotics—live microbial strains—demonstrating potential to modulate DC function and Treg induction (Yao et al. 2023; Ahmadi‐Khorram et al. 2025). *Lactobacillus rhamnosus* and *Lactobacillus delbrueckii* have shown anti‐inflammatory effects, shifting the Treg–Th17 balance toward immune tolerance in autoimmune disease models (Esmaeili et al. 2017; Shi et al. 2024; Fong et al. 2015; Rocha et al. 2014, 2012). A 2023 systematic review further underscored the role of probiotics in reducing inflammation and improving immune responses in SLE (Mirfeizi et al. 2023). Building on our previous work, which demonstrated that *L. rhamnosus* and *L. delbrueckii* induce tolerogenic DCs with upregulated indoleamine 2,3‐dioxygenase (IDO) expression and reduced co‐stimulatory markers (Esmaeili et al. 2018), this study evaluates their therapeutic potential in modulating Treg, Th1, and Th17 responses to restore immune balance in an in vitro SLE model.

## 2 Methods

### 2.1 Reagents, Materials and Bacterial Preparation

*Lactobacillus delbrueckii* subsp. lactis (PTCC: 1743; DSM 20072) and *Lactobacillus rhamnosus* (ATCC: 9595) were obtained from the Iranian Research Organization for Science and Technology (IROST) and the Pasteur Institute of Iran, respectively. Bacterial strains were cultured in de Man, Rogosa, and Sharpe (MRS) broth (Biolife, Italy) under microaerobic conditions at 37°C for 1 h, followed by plating on MRS agar for 24 h. Colonies were stored as glycerol stocks at −70°C. Additional reagents included Ficoll–Hypaque (Sigma‐Aldrich), RPMI‐1640 medium, penicillin/streptomycin, fetal bovine serum (FBS), lipopolysaccharides (LPS; Sigma‐Aldrich), L‐glutamine, TRIzol reagent (Invitrogen), reverse transcriptase (Fermentas), and SYBR Green PCR kit (Qiagen).

### 2.2 Peripheral Blood Mononuclear Cells (PBMCs) Isolation

Venous blood samples (15 mL) were collected in heparinized tubes from SLE patients and healthy controls. PBMCs were isolated by density gradient centrifugation using Ficoll–Hypaque. The interface layer was harvested, washed twice with phosphate‐buffered saline (PBS, pH 7.4), and resuspended in RPMI‐1640 medium supplemented with 10% FBS, 100 U/mL penicillin, 100 μg/mL streptomycin, and 2 mM L‐glutamine for subsequent experiments.

### 2.3 Dendritic Cell (DC) Generation and Probiotic Treatment

Immature DCs were generated from monocytes isolated from peripheral blood mononuclear cells (PBMCs) using CD14+ magnetic‐activated cell sorting (MACS; Miltenyi Biotec) and treated with *Lactobacillus rhamnosus* , *Lactobacillus delbrueckii* , a combination of both probiotics (1:1 ratio, 10^8^ CFU/mL), or LPS (1 μg/mL) as a pro‐inflammatory control, as previously described (Esmaeili et al. 2018). DC maturation was confirmed by flow cytometry, assessing CD11c, HLA‐DR, and CD86 expression.

### 2.4 T Cell Isolation and Culture

Naive CD4+ T cells were isolated from PBMCs using negative selection MACS kits (Miltenyi Biotec) after a 2‐h incubation at 37°C. T cells (1 × 10^6^ cells/well) were cultured in 24‐well plates with RPMI‐1640 medium supplemented with 10% FBS. For Treg induction, cells were stimulated with plate‐bound anti‐CD3 (5 μg/mL) and soluble anti‐CD28 (2 μg/mL) antibodies (eBioscience) for 5–7 days at 37°C with 5% CO_2_.

### 2.5 Mixed Lymphocyte Reaction (MLR)

To assess the impact of probiotic‐treated DCs on T cell differentiation, naive CD4+ T cells were co‐cultured with treated DCs at a 4:1 ratio in 24‐well plates containing RPMI‐1640 medium with 10% FBS. Cultures were incubated at 37°C with 5% CO_2_ for 5 days to allow DC–T cell interactions. LPS‐treated DCs served as the control.

### 2.6 RNA Extraction and cDNA Synthesis

According to the manufacturer's protocol, total RNA was extracted from T cells (0.2 × 10^6^) using Tripura reagent. Complementary DNA (cDNA) was synthesized by combining 1 μg of total RNA, a 100 mM random hexamer primer, and reverse transcriptase enzyme (200 U/mL; Fermentas).

### 2.7 Flow Cytometry Analysis

T cells (1 × 10^6^) from MLR cultures were stained with FITC‐conjugated anti‐CD4, APC‐conjugated anti‐CD127, and PE‐conjugated anti‐CD25 antibodies (eBioscience) for 30 min at 4°C in the dark. After fixation and permeabilization (eBioscience Fix/Perm buffer), cells were stained with PE‐Cy5‐conjugated anti‐FOXP3 for Treg identification or FITC‐conjugated anti‐IL‐17 and PE‐conjugated anti‐IFN‐γ for Th17 and Th1 cells, respectively. Isotype controls ensured specificity. Data were acquired on a FACSCalibur flow cytometer (Becton Dickinson) and analyzed using FlowJo v7.6.2 (Tree Star). Cell populations were gated based on size, granularity, and marker expression.

### 2.8 RNA Extraction and cDNA Synthesis

Total RNA was extracted from 0.2 × 10^6^ T cells using TRIzol reagent according to the manufacturer's protocol. Complementary DNA (cDNA) was synthesized from 1 μg RNA using 100 mM random hexamer primers and 200 U/mL reverse transcriptase (Fermentas) in a 20 μL reaction volume.

### 2.9 Real‐Time Quantitative PCR

Gene expression of FOXP3, TGF‐β, IL‐10, ROR‐γt, IL‐17, T‐bet, IFN‐γ, GATA3, and IL‐4 was quantified using SYBR Green PCR (Qiagen) on a Rotor‐Gene 6000 thermal cycler (Qiagen). Glyceraldehyde‐3‐phosphate dehydrogenase (GAPDH) served as the internal control. Primer sequences were validated for specificity, and melting curve analysis confirmed product specificity. Relative gene expression was calculated using the 2^‐ΔΔCt method.

### 2.10 Statistical Analysis

Data were analyzed using SPSS v11.5 (IBM) and GraphPad Prism v5 (GraphPad Inc.). Differences between groups were assessed using one‐way analysis of variance (ANOVA) with post hoc Tukey tests. A *p* value ≤ 0.05 was considered statistically significant. Data are presented as mean ± standard deviation (SD).

## 3 Results

### 3.1 Study Participants

The study included three female patients newly diagnosed with systemic lupus erythematosus (SLE) (mean age: 35 ± 3 years) and three age‐matched healthy female controls (mean age: 33 ± 2 years). All SLE patients were positive for antinuclear antibodies (ANA) and anti‐double‐stranded DNA (anti‐dsDNA) antibodies. Monocyte purity, isolated from peripheral blood mononuclear cells (PBMCs), was confirmed by flow cytometry to be > 95% CD14+.

### 3.2 Treg Cell Analysis (CD4 ^+^ CD25 ^+^ CD127 ^−^ FOXP3 ^+^)

**FIGURE 1:**
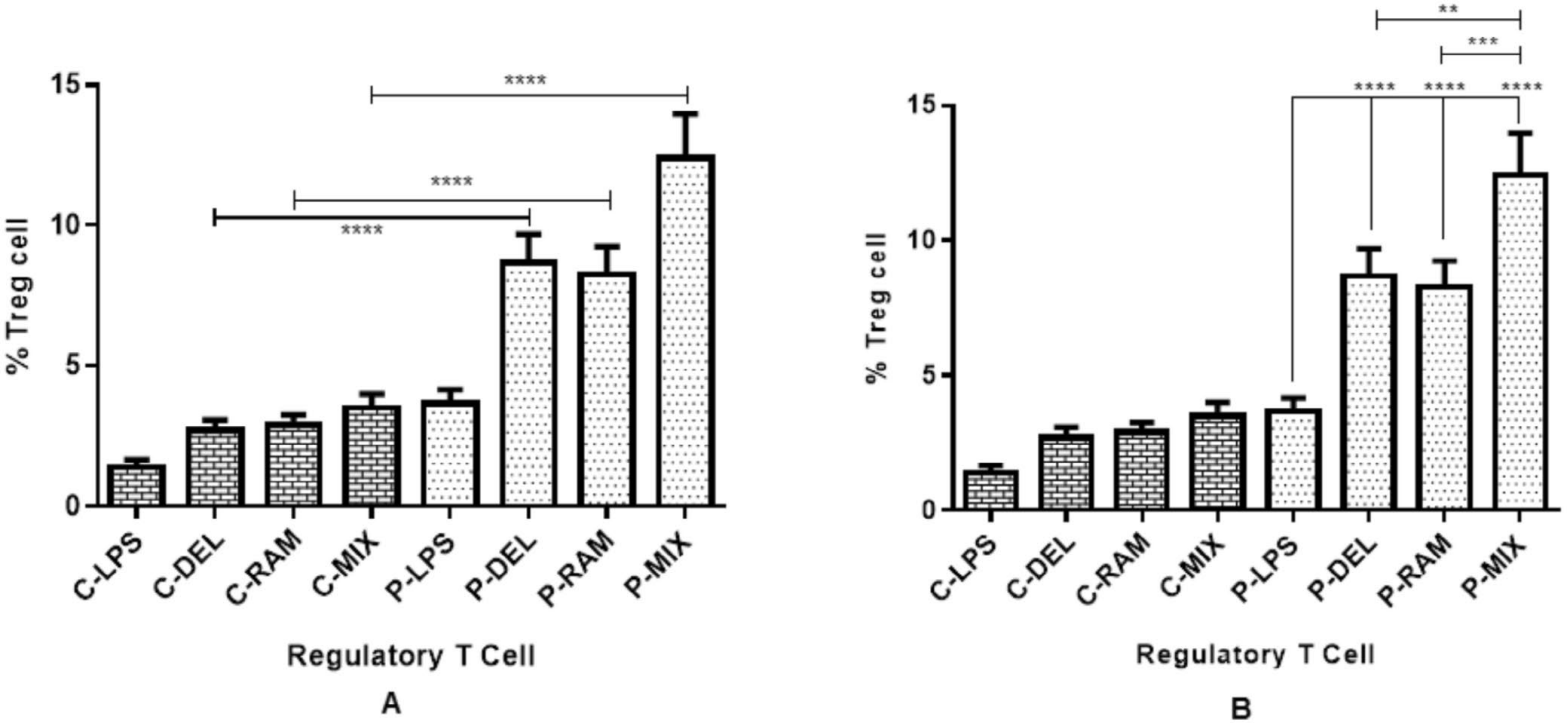
Treg cell frequencies in co‐cultures of naive T cells with DCs treated with probiotics or LPS in healthy controls and SLE patients. (A) SLE patients showed significantly higher Treg frequencies than healthy controls. (B) In SLE patients, probiotic‐treated groups (P‐DEL, P‐RAM, P‐MIX) exhibited significantly higher Treg frequencies than the LPS group, with P‐MIX showing the greatest effect (*p = 0.001, **p = 0.0004, ***p ≤ 0.0001). Data were analyzed by one‐way ANOVA.

Flow cytometry revealed significantly higher frequencies of CD4^+^CD25^+^CD127^−^FOXP3^+^ regulatory T cells (Tregs) in SLE patients compared to healthy controls (*p* < 0.01). Probiotic‐treated dendritic cells (DCs) significantly enhanced Treg frequencies in SLE patients compared to the lipopolysaccharide (LPS)‐treated group (*p* ≤ 0.0001). The combined *Lactobacillus rhamnosus* and *L. delbrueckii* treatment (P‐MIX) induced the highest Treg frequencies in SLE patients, significantly surpassing *L. delbrueckii* alone (P‐DEL, *p* = 0.001) and *L. rhamnosus* alone (P‐RAM, *p* = 0.0004) (Figure 1A). In healthy controls, probiotic‐treated groups showed increased Treg frequencies compared to the LPS group, but differences were not statistically significant (Figure 1B).

### 3.3 Th17 Cell Analysis (CD4 ^+^ IL‐17^+^)

**FIGURE 2:**
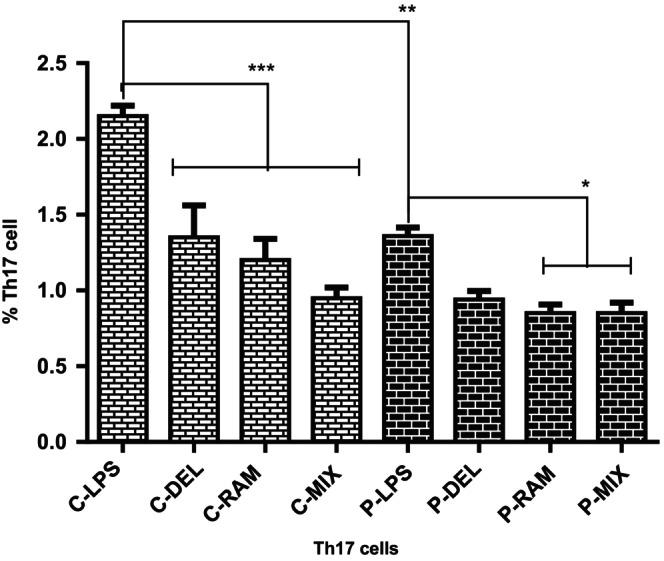
Th17 cell frequencies in co‐cultures of naive T cells with probiotic‐ or LPS‐treated DCs. Probiotic‐treated groups in both healthy controls and SLE patients showed reduced Th17 frequencies compared to LPS‐treated groups. LPS‐treated SLE patients had lower Th17 frequencies than LPS‐treated healthy controls (*p = 0.037, **p = 0.018, ***p = 0.0005, ****p ≤ 0.0001). Data were analyzed by one‐way ANOVA.

Th17 cell frequencies (CD4^+^IL‐17^+^) were assessed by flow cytometry after co‐culture with probiotic‐ or LPS‐treated DCs. In healthy controls, probiotic‐treated groups ( *L. delbrueckii* [C‐DEL], *L. rhamnosus* [C‐RAM], and combined [C‐MIX]) showed significantly reduced Th17 frequencies compared to the LPS group (*p* = 0.018, *p* = 0.0005, and *p* ≤ 0.0001, respectively). In SLE patients, Th17 frequencies were significantly lower in the P‐RAM and P‐MIX groups compared to the LPS group (*p* = 0.037), with no significant difference between P‐DEL and LPS. Notably, Th17 frequencies in LPS‐treated SLE patients were significantly lower than in LPS‐treated healthy controls (*p* = 0.002). No significant differences were observed between probiotic‐treated SLE patients and healthy controls (Figure 2).

### 3.4 Th1 Cell Analysis (CD4 ^+^ IFN‐γ^+^)

**FIGURE 3:**
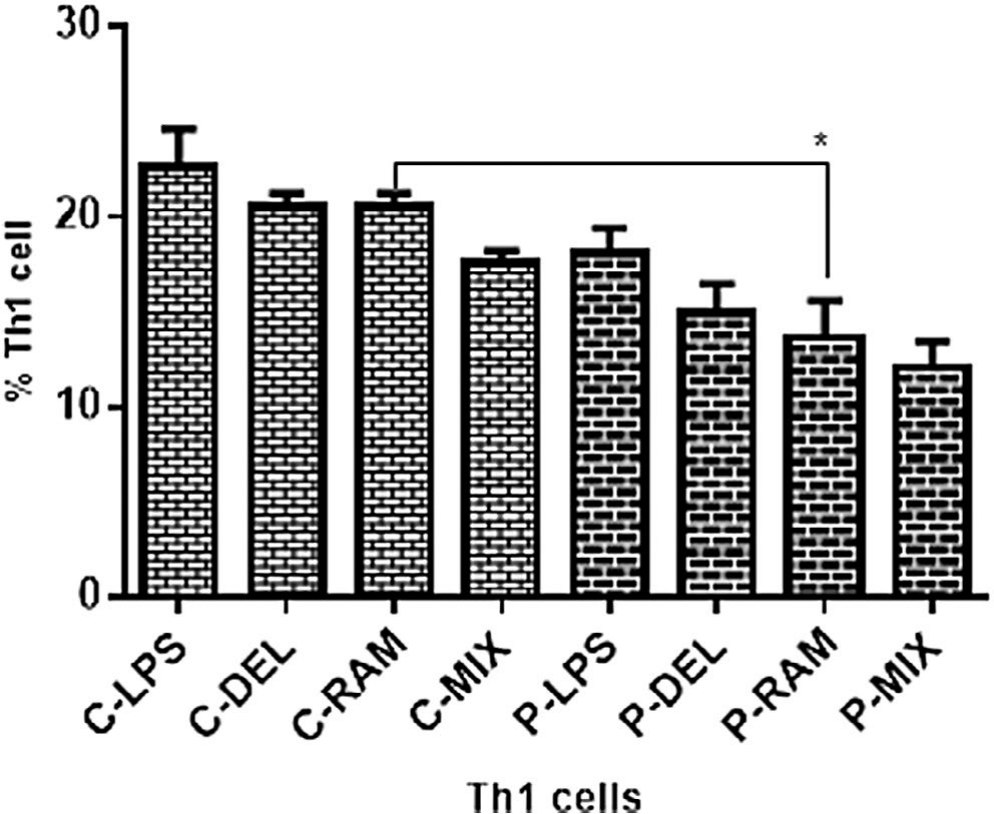
Th1 cell frequencies in co‐cultures of naive T cells with probiotic‐ or LPS‐treated DCs. Probiotic‐treated groups showed reduced Th1 frequencies compared to LPS‐treated groups, with a significant reduction in P‐RAM vs. C‐RAM (*p = 0.0315). Data were analyzed by one‐way ANOVA.

Probiotic treatment reduced CD4^+^IFN‐γ^+^ Th1 cell frequencies in both SLE patients and healthy controls compared to the LPS group, though these reductions were not statistically significant except in the P‐RAM group compared to the C‐RAM group (*p* = 0.0315). No significant differences were observed between SLE patients and healthy controls across other groups (Figure 3).

### 3.5 Gene Expression

#### 3.5.1 FOXP3, TGF‐β, and IL‐10 Expression

**FIGURE 4:**
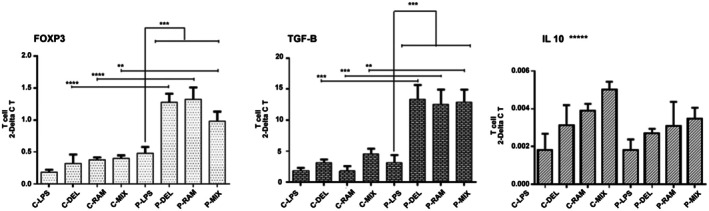
Relative gene expression of FOXP3, TGF‐β, and IL‐10 in T cells. In SLE patients, probiotic‐treated groups showed significantly higher FOXP3 and TGF‐β expression compared to healthy controls and the LPS group (*p ≤ 0.05, **p ≤ 0.0081, ***p ≤ 0.0005, ****p ≤ 0.0001). IL‐10 expression was elevated but not significant (*****p > 0.05). Data were analyzed by one‐way ANOVA.

In SLE patients, probiotic‐treated DCs significantly upregulated FOXP3 expression compared to healthy controls (P‐DEL: *p* ≤ 0.0001; P‐RAM: *p* ≤ 0.0001; P‐MIX: *p* = 0.0081) and the LPS group (*p* ≤ 0.05). Similarly, TGF‐β expression was significantly higher in SLE probiotic‐treated groups compared to healthy controls (P‐DEL: *p* = 0.0004; P‐RAM: *p* = 0.0007; P‐MIX: *p* = 0.0045) and the LPS group (*p* ≤ 0.0005). IL‐10 expression was elevated in all probiotic‐treated groups compared to the LPS group in both SLE patients and healthy controls, but these increases were not statistically significant (Figure 4).

#### 3.5.2 ROR‐γt and IL‐17 Expression

**FIGURE 5:**
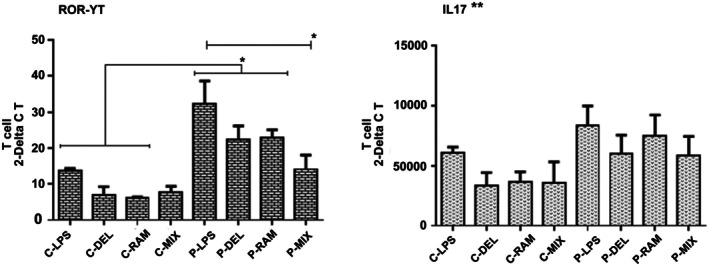
Relative gene expression of ROR‐γt and IL‐17 in T cells. ROR‐γt expression was higher in SLE patients, with P‐MIX significantly reducing expression compared to the LPS group (*p ≤ 0.05). IL‐17 expression was higher in SLE patients but not significantly reduced in probiotic‐treated groups (**p > 0.05). Data were analyzed by one‐way ANOVA.

ROR‐γt expression was significantly higher in SLE patients than in healthy controls (LPS: *p* = 0.0123; P‐DEL: *p* = 0.0367; P‐RAM: *p* = 0.0233). In SLE patients, the P‐MIX group showed a significant reduction in ROR‐γt expression compared to the LPS group (*p* = 0.0136). No significant differences were observed among healthy control groups. IL‐17 expression was higher in SLE patients than in healthy controls, with reductions in probiotic‐treated groups compared to the LPS group, though these were not statistically significant (Figure 5).

#### 3.5.3 T‐Bet and IFN‐γ Expression

**FIGURE 6:**
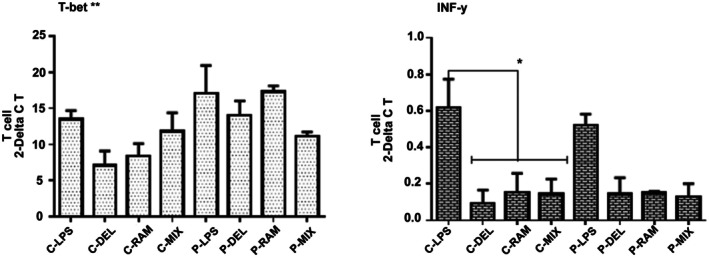
Relative gene expression of T‐bet and IFN‐γ in T cells. IFN‐γ expression was significantly reduced in probiotic‐treated healthy controls compared to the LPS group (*p ≤ 0.05). No significant differences were observed in T‐bet expression (**p > 0.05). Data were analyzed by one‐way ANOVA.

T‐bet expression showed no significant differences across groups. In healthy controls, probiotic‐treated groups exhibited reduced IFN‐γ expression compared to the LPS group (C‐DEL: *p* = 0.0105; C‐RAM: *p* = 0.0235; C‐MIX: *p* = 0.0207). In SLE patients, IFN‐γ expression was lower in probiotic‐treated groups compared to the LPS group, but these reductions were not statistically significant (Figure 6).

#### 3.5.4 GATA3 and IL‐4 Expression

**FIGURE 7:**
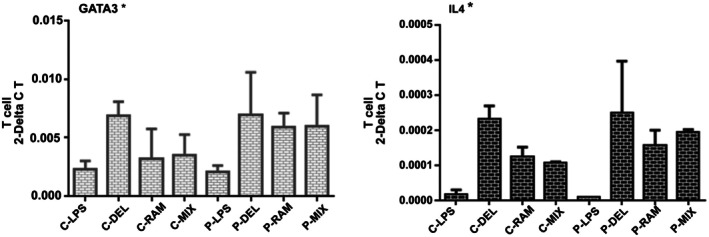
Relative gene expression of GATA3 and IL‐4 in T cells. No significant differences were observed between groups (*p > 0.05). Data were analyzed by one‐way ANOVA.

No significant differences were observed in GATA3 or IL‐4 expression between SLE patients and healthy controls. Probiotic‐treated groups showed a trend toward increased GATA3 and IL‐4 expression compared to the LPS group in both cohorts, but these changes were not statistically significant (Figure 7).

## 4 Discussion

This study demonstrates that *Lactobacillus rhamnosus* and *Lactobacillus delbrueckii* induce tolerogenic dendritic cells (DCs) that enhance regulatory T cell (Treg) induction and suppress pro‐inflammatory Th17 and Th1 responses in systemic lupus erythematosus (SLE). Building on our prior work showing upregulated indoleamine 2,3‐dioxygenase (IDO) expression and reduced co‐stimulatory markers in probiotic‐treated DCs (Esmaeili et al. 2018; Babazadeh et al. 2025; Ahmadi et al. 2025), we found that these DCs significantly increase Treg frequencies (Figure 1A) through elevated FOXP3 and TGF‐β expression while reducing Th17 and Th1 populations in SLE patients (Figures 2 and 3). The combined *L. rhamnosus* and *L. delbrueckii* treatment (P‐MIX) exhibited the most pronounced Treg induction (*p* = 0.0004, Figure 1A), suggesting that multi‐strain probiotics may offer superior therapeutic benefits for autoimmune diseases.

The significant increase in Treg populations and associated FOXP3 and TGF‐β expression in SLE patients (Figure 4) aligns with prior studies. Rocha et al. (2014) reported enhanced Treg induction in colitis models treated with *L. delbrueckii* , while Karimi et al. (2012) showed that *L. rhamnosus* upregulated FOXP3+ Tregs in gut‐associated lymphoid tissue. Our findings extend these observations to SLE, suggesting that probiotic‐induced tolerogenic DCs modulate T cell differentiation through FOXP3 and TGF‐β pathways. The lack of significant IL‐10 elevation (Figure 4), despite a trend toward increased expression, may indicate that *L. rhamnosus* and *L. delbrueckii* primarily enhance Treg activity via FOXP3 and TGF‐β rather than IL‐10. This contrasts with studies like van der Kleij et al. (2008), which reported IL‐10 induction by *L. rhamnosus* in intestinal T cells, potentially reflecting disease‐specific immune dysregulation in SLE or differences in experimental models, such as in vitro versus in vivo settings.

Probiotic‐treated DCs significantly reduced Th17 cell frequencies and ROR‐γt expression (Figure 5), particularly in the P‐MIX group (*p* = 0.0136), highlighting their ability to suppress pro‐inflammatory responses critical in SLE (Dolff et al. 2011; Mohamed et al. 2025). These findings are consistent with Chen et al. (2016), who demonstrated that *L. rhamnosus* balances Treg and Th17 populations in autoimmune models. The pronounced Th17 suppression in SLE patients compared to healthy controls suggests heightened responsiveness of SLE‐derived DCs, possibly due to elevated IDO expression (Esmaeili et al. 2018). The nonsignificant reduction in IL‐17 expression (Figure 5) may reflect contributions from non‐T cell sources, such as innate lymphoid cells (Arpaia et al. 2013), or the need for larger sample sizes.

Probiotic treatment also reduced Th1 cell frequencies and IFN‐γ expression (Figure 6), with a significant reduction in the P‐RAM group compared to C‐RAM (*p* = 0.0315). Although not statistically significant in SLE patients, this trend aligns with reports of probiotic‐mediated IFN‐γ suppression (Fong et al. 2015). The lack of significant T‐bet changes suggests that probiotics may preferentially modulate cytokine production rather than Th1 differentiation, relevant in SLE where IFN‐γ drives autoantibody production (Kwon et al. 2010; Nandakumar and Nündel 2022). No significant changes in GATA3 or IL‐4 expression (Figure 7) indicate a limited impact on Th2 responses, consistent with the Th1/Th17‐driven pathology of SLE, where Th2 modulation is less critical (Dolff et al. 2011). However, the slight increase in GATA3 and IL‐4 in probiotic‐treated groups suggests potential subtle effects on Th2 pathways, which warrant further investigation, particularly in contexts where Th2 responses may play a secondary role, such as in lupus nephritis (An et al. 2022).

The synergistic effect of combined probiotics likely arises from complementary mechanisms, such as enhanced DC tolerogenicity and cytokine modulation (Kwon et al. 2010). Recent evidence suggests microbial metabolites, like short‐chain fatty acids (SCFAs), may enhance Treg differentiation and suppress inflammation (Smith et al. 2013). Notably, a 2025 review on immune‐metabolic restoration in SLE demonstrated that multi‐strain Lactobacillus probiotics significantly enhance Treg/Th17 balance and reduce renal inflammation through SCFA‐mediated pathways, providing compelling clinical rationale for the translational potential of our P‐MIX strategy (Habiballah et al. 2025).

### 4.1 Limitations and Future Directions

The small sample size (three SLE patients and three healthy controls) limits the generalizability of our findings, necessitating larger cohort studies to validate these results. Additionally, the in vitro nature of our experiments may not fully capture the complex in vivo interactions between probiotics, gut microbiota, and systemic immunity. Future studies should explore the clinical efficacy and safety of *L. rhamnosus* and *L. delbrueckii* in SLE patients, including longitudinal trials to assess their impact on disease activity and gut microbiome composition. Investigating the molecular mechanisms underlying probiotic‐induced DC tolerogenicity, such as epigenetic modifications or microbial metabolite signaling, could further elucidate their therapeutic potential.

## 5 Conclusions

This study provides novel insights into the immunomodulatory effects of *L. rhamnosus* and *L. delbrueckii* in SLE, demonstrating their ability to promote tolerogenic DCs and Treg induction while suppressing Th17 and Th1 responses. The synergistic effects of combined probiotic treatment highlight their potential as a safe, complementary therapy for managing immune dysregulation in SLE. These findings pave the way for further clinical research to establish probiotics as a viable therapeutic strategy for autoimmune diseases.

## Author Contributions

M.A.‐K., A.H., A.F., and R.K.K. participated in data collection, project performance, and manuscript writing. M.M. and S.‐A.E. designed and drafted the article. All authors have read and approved the final manuscript.

## Funding

The authors have nothing to report.

## Conflicts of Interest

The authors declare no conflicts of interest.

## Data Availability

The data that support the findings of this study are available from the corresponding author upon reasonable request.
